# A POOL OF INTERESTED PARTIES: COMMUNICATION, ENTRYWAYS, AND USER INVOLVEMENT IN AGING RESEARCH IN A SWEDISH CONTEXT

**DOI:** 10.1093/geroni/igae098.1296

**Published:** 2024-12-31

**Authors:** Oskar Jonsson, Susanne Iwarsson, Steven Schmidt

**Affiliations:** Lund University, Lund, Skane Lan, Sweden; Lund University, Lund, Skane Lan, Sweden; Lund University, Lund, Skane Lan, Sweden

## Abstract

Established in 2007, the Centre for Ageing and Supportive Environments (CASE) is an interdisciplinary research environment at Lund University, Sweden. The center’s activities are led by a steering committee in close collaboration with the CASE User Council, which provides input, ideas and poses research questions for various research projects. Between 2018-2024, CASE was responsible for one of Lund University’s thematic collaboration initiatives – Social Rights and Housing for the Ageing Population, which is a partnership for knowledge mobilization contributing to solutions for key societal challenges. As one forward-looking way to prolong the established initiative with a long-term ambition, we have launched a national registry, which will be used as a pool of interested parties with the aim to raise awareness about aging research and the possibility to engage in research, mobilize knowledge and utilize research results. The approach is to offer people with an interest in research on aging and health to register in a nationwide online person register/database to systematize and facilitate communication, entryways to research studies, and user involvement. The development of the pool has been fraught with issues related to terminology, ethics, legal requirements for handling personal data, and providing necessary information without being overwhelming but instead attractive for the diverse individuals and groups within the civil society, business, and public authorities that we target. This presentation will provide an overview of the multiple strategies and tactics we employ to tailor the information flow and interactive process based on individual and group-specific needs and preferences.

